# Mental Health Stigma in the Workplace and its Association with Possible Actions of Managers to Prevent Sickness Absence of Employees with Mental Health Problems in the Swedish Private Sector: a Video Vignette Study

**DOI:** 10.1007/s10926-024-10220-z

**Published:** 2024-07-11

**Authors:** Sofie Schuller, Angelique de Rijk, Linda Corin, Monica Bertilsson

**Affiliations:** 1 Department of Public Administration and Sociology, School of Social and Behavioural Sciences, Erasmus University, PO Box 1738, 3000 DR Rotterdam Rotterdam, The Netherlands; 2 Department of Social Medicine, Faculty of Health, Medicine and Life Sciences, Primary Care and Public Health Research Institute, Maastricht University, Maastricht, The Netherlands; 3 Institute of Stress Medicine, Region Västra Götaland, Gothenburg, Sweden; 4 School of Public Health and Community Medicine, Institute of Medicine, The Sahlgrenska Academy, University of Gothenburg, Gothenburg, Sweden

**Keywords:** Mental health, Social stigma, Workplace, Sick leave, Prevention

## Abstract

**Purpose:**

Mental health problems (MHPs) are subjected to workplace stigma and can deteriorate into common mental disorders (CMDs) and sickness absence (SA). Research has shown that personal stigmatizing attitudes limit managers’ efforts towards employees with MHPs, but knowledge is lacking regarding stigma in social contexts (contextual stigma) and different types of possible preventive actions. This study investigates personal stigmatizing attitudes and three contextual stigma layers (employee, collegial, organizational) and different types of possible actions to prevent SA of employees with MHPs.

**Method:**

Survey data of 2769 Swedish managers working in the private sector were analysed. Personal stigmatizing attitudes were measured with the managerial stigma towards employee depression scale and supplemented with four additional items capturing contextual stigma. Managers watched video vignettes and assessed which preventive actions (*n*  = 20) were possible to use in their organization. A sum score was calculated reflecting the ‘number of actions’. Principal component analysis revealed three action types: adapt tasks and setting, involve experts, and social support. A score reflecting the ‘possibilities to implement actions’ was calculated for each type. Multiple linear regression analyses were conducted with the four stigma layers as independent variables for each of the three action variables.

**Results:**

Personal stigmatizing attitudes and contextual stigma were significantly associated with both ‘number of actions’ and ‘possibilities for implementing actions’ relating to all action types. Patterns of associations with contextual stigma were significant but varied between the different action types.

**Conclusion:**

This study substantiated the role of personal stigmatizing attitudes and contextual stigma in relation to possible actions of managers to prevent SA of employees with MHPs. The results emphasize the role of contextual stigma. Implications for practice and research are discussed.

**Supplementary Information:**

The online version contains supplementary material available at 10.1007/s10926-024-10220-z.

## Introduction

Mental health problems (MHPs) are a widespread issue among the working age population in western countries [1–4]. MHPs have an adverse impact on work performance and capacity, and can deteriorate into common mental disorders (CMDs), today the leading diagnosis for sickness absence (SA) [5, 6]. Consequentially, companies face considerable costs [5], possible strain on other employees [6], and possible complications for return to work (RTW) efforts [7]. MHP is an umbrella term, that compared to CMD, includes subclinical and subthreshold mental health conditions [4]. The role of the workplace is two-fold when it comes to MHPs; psychologically strenuous working conditions can be the source of, or contribute to MHPs [4, 8], whereas staying at work can be beneficial for the recovery and well-being of employees with MHPs [9]. Thus, active efforts of managers to support the employee at work are crucial, as interventions show that managerial actions to alleviate stress, adapt the work or workplace, and complement the employee’s capacity can help the employee to stay at work [10, 11]. However, managers describe several barriers to taking action, such as a lack of knowledge of MHPs, difficulties understanding the impact of MHPs on work capacity, as well as stigma and prejudice [12, 13]. The impact of these barriers is substantiated by research revealing a problematic lack of action taken towards employees with MHPs. For example, reviewing the work assignments and work situation aids in identifying possible stressors for employees, however a Swedish study investigating actions to prevent MHPs showed that only 51.6% of managers reviewed the work assignments and work situation within the past two years [14]. Similarly, an investigation of Danish managers revealed that only 51% of the managers actively approached employees with depression to offer help [15].

Stigma in the workplace affects employees with MHPs in several ways: mental health stigma in the workplace has been shown to lower the likelihood of employees self-disclosing mental health issues [16], puts them at risk of receiving less work accommodations compared to employees with physical complaints [17, 18], and contributes to perceptions of CMDs as a less valid reason for SA [19].

Asking managers what they find helpful when taking action for employees with MHPs, they emphasized awareness of mental health and supportive company policies as facilitating factors [20]. Building a healthy workplace can further aid the prevention of MHPs and SA with a CMD. Research into mental health programmes and interventions shows that both experts and employees call for destigmatization efforts [21, 22]. Destigmatizing mental health would facilitate affected employees to seek help, as well as support the implementation of further mental health programmes [21, 22]. Overall, stigma has been identified as a major barrier to handling MHPs in the workplace by managers, experts, and employees.

When accommodating MHPs at work, managers more often provided flexible scheduling, but less often modified work duties or adapted the working environment [23]. In a more recent study regarding RTW efforts, managers predominantly reported having made changes to the work schedule, tasks, and environment, but managers rarely involved colleagues or occupational health services [24]. In line with the varied prevalence of different types of actions, it has been shown that the different managerial actions vary according to personal factors of the manager (e.g., gender and work experience) as well as contextual factors (e.g., stress counselling at the workplace) [14, 25]. Thus, it is appropriate to distinguish different action types in SA prevention.

The low level of managerial action might also largely be connected to the attached mental health stigma. Several studies have established the negative impact managers’ personal stigmatizing attitudes towards mental health issues can have for employees with MHPs [16, 18, 26, 27]. Here, research on stigma emphasizes the need to examine manager’s social context and influences as a route to improve the situation for employees with MHPs [26, 27]. Brouwers and colleagues [28] illustrate the role of social stigma in unemployment of individuals with MHPs, where social stigma is defined on three levels; 1) individual stigma referring to a person’s self-stigmatization, 2) interpersonal stigma occurring in the interaction between stigmatized and non-stigmatized individuals, and 3) structural stigma referring to stigmatization that has been institutionalized in law and policy on a societal level. Within organizations, structural stigma could be reflected in company policies and norms which can limit resources and possibilities for managers to support employees with MHPs. Workplace structural stigma constitutes contexts affecting all populating the workplace, and also relates to public stigma. Public stigma describes the stigmatizing attitudes and behaviours of a general population [29]. Public stigma is anchored in the sense of community and culture shared within a group of people. Applied to the work context, public could be defined as the body of individuals working in an organization, sharing community and culture within the workplace. Public stigma is assumed to influence the behaviour of different individuals and groups, such as managers and other employees, and might lead to differential treatment and discrimination. In summary, we argue for a layered understanding of social stigma in the workplace. Structural stigma is reflected in discriminatory company policies on an organizational level while different social groups, such as employees and managers, enact their public stigma through differential treatment.

More concretely, stigma does in fact impact managers’ possibilities to take action in preventing the SA of employees with MHPs. Low personal stigmatizing attitudes of managers were shown to be the second most important determinant for staying in touch with employees on SA [30]. Correspondingly, stigmatization of mental health is associated with negative outcomes for individuals with MHPs. Co-workers’ and managers’ beliefs that mental health is rooted in a person’s behaviour correlated with less managerial action and co-worker support [31, 32], illustrating how personally held attitudes and public stigma are hindrances to taking action.

These findings illustrate the relevance of stigma for improving the workplace and work situation for employees with MHPs, as well as the pivotal role of managers in these efforts. Further, researchers recommend exploring contextual factors in relation to managerial actions, as well as the role of different stigma layers in this relationship [27, 33, 34]. Based in both workplace structural stigma and public stigma, this study identifies four layers of stigma in relation to managers: personal stigmatizing attitudes; public—among employees, and among managerial colleagues; and structural within the organization. Similar structures of layered social contexts are employed to explain experienced gender differences, emphasizing the added insight and nuance provided by the consideration of social contexts when examining the mechanisms of differential treatment [35].

In conclusion, this study aims to investigate the association between the different stigma layers and managers’ possibilities to take action to prevent the SA of employees with MHPs. The following aspects are examined:

1. To what extent are the different stigma layers (personal, employee, collegial, organizational) regarding MHPs associated with managers’ assessment of the number of actions they can take to prevent SA of employees with MHPs?

2. To what extent are the different stigma layers (personal, employee, collegial, organizational) regarding MHPs associated with managers’ assessments of possibilities to implement preventive actions in relation to SA of employees with MHPs?

## Methods

### Study Design

This study made use of data from the managers in action research project at the University of Gothenburg (AFA Dnr: 190, 130). In this project, the importance of organizational contextual factors for the prevention of SA of employees with MHPs was investigated from the perspective of private sector managers. Data were collected using an online survey assessing aspects of organizational structure, work context, organizational climate, job demands and resources, and managers’ personal characteristics. To assess possible managerial actions to prevent SA in relation to realistic employee cases, a video vignette design was employed. The survey was piloted on 12 managers before the start of data collection. The managers in action project was approved by the Swedish ethical review board (Dnr: 2021–02808).

### The Swedish Setting

The managers in action project is tailored to the Swedish private sector’s working context, legislation, and culture. Employers are mandated to invest in employee well-being and efforts to prevent SA per Swedish legislation [36]. This puts managers in a central position of responsibility because they are in the position of having a direct influence on the employee’s situation at work [36]. Further, the Swedish context has a proactive approach to mental health stigma. For example, since 2009 the Swedish government raises awareness for mental health through an ambassador programme, including courses on how managers and colleagues can support individuals with MHPs [37].

### Participants

Participants were approached through the Swedish citizen panel at the SOM institute (*n* = 75,000), University of Gothenburg, resulting in a self-recruited convenience sample. Invitations to take part in the survey were sent in 2021 to 5654 individuals listed in the panel as private sector managers. The study population included 4091 individuals who met the following criteria: (a) holding a managerial or supervisory position, (b) being employed in the private sector, and (c) having responsibility for subordinates. Participants were asked to confirm that they met the criteria and to provide informed consent at the beginning of the questionnaire; the survey terminated if the criteria were not met, or consent was not given. An additional inclusion criterion for this study was to have seen the video vignette within the survey. The final study sample consisted of 2769 managers. There was an internal dropout of 709 participants.

### Measures

#### The Independent Variables

Personal stigmatizing attitudes were measured using an adapted version of the managerial stigma towards employee depression (MSED) scale [38], consisting of three subscales assessing affective, cognitive, and behavioural stigma. The instrument was translated into Swedish for previous research [16, 39]. Further adaptations were made to the questions to reflect several CMDs (anxiety, depression, exhaustion). The revised items of the MSED are shown in Supplementary Table 1. The scale maintained an acceptable internal consistency (Cronbach’s *α* = 0.80). Response options ranged from (1) ‘do not agree at all’ to (6) ‘agree completely’. An MSED score was calculated for each participant. The contextual stigma measures were designed for this project based on previous research [27, 39] and introduced with the question: ‘To what extent do you think the following statements apply in your company?’. Employee stigma was assessed by two items (‘Most employees in our organization would talk about exhaustion, anxiety, or depression at work’; ‘My work group accepts me bringing up issues relating to exhaustion, anxiety, or depression in joint discussions’). A mean score was calculated. Collegial stigma was assessed using a single item (‘In our company, it is acceptable among managers and supervisors to talk about employees’ exhaustion, anxiety, or depression’). Organizational stigma was assessed using a single item (‘In our company, it is acceptable to talk about exhaustion, anxiety, and depression’). The response options for the employee, collegial, and organizational stigma measures ranged from (1) ’do not agree at all’ to (5) ’very much agree’, including a ’no opinion’ option (6). The ‘no opinion’ option was added to prevent socially desirable answers. The frequency of use of this option for each of the items was below 7%, therefore ‘no opinion’ responses were excluded from the analysis without significant loss of data.

#### The Dependent Variables Based on Video Vignettes

Each participant answered questions regarding possible preventive actions in relation to one of six vignettes. These video vignettes were developed to represent three industrial sector settings (blue—working with things; white—working with symbols; pink—working with humans) and two genders of employees (female and male), based on a common distinction [40]. A detailed description of the industrial sectors is given in Supplementary Table 2. A one-minute video was recorded for each industrial sector, with a female and with a male employee as protagonists. In the video, the employee tells their manager about problems keeping up with their workload due to MHP-related issues (Supplementary Table 3). Participants were presented with a vignette corresponding to their relevant industrial sector, and randomly depicting a female or male employee. In cases of participants working in an ‘other’ industrial sector, they were shown a vignette set in the white sector. Participants then indicated their possibility to implement each of 20 preventive actions in relation to the presented vignette. These 20 actions had previously been identified in a study using eight qualitative focus groups to explore managers' experience-based understanding of the capacity to work of employees with MHPs [41] and the managers' strategies to support these employees (in manuscript). A total of 31 managers took part in these focus groups. The items were introduced with: ‘The following questions relate to how employees with exhaustion, anxiety or depression can get support in your workplace. Imagine that this person in the video is your employee from whom you are the manager. On the following pages, you will answer questions about possible actions that you can take at your workplace to avoid him/her becomes sick-listed?’. After watching the video vignette, each action was evaluated using the question ‘At your workplace, to what extent is it possible for you as manager to:’. Response options were (1) ‘not at all’, (2) ‘possible to a certain extent’, (3) ‘possible to a great extent’, and (4) ‘fully possible’.

The number of actions was calculated by summing the dichotomized responses to the action items (‘not possible’ (1, score 0) and ‘possible’ (2, 3, 4, score 1)). The possibility for implementing actions was operationalized using principal component analysis to reduce the 20 actions into fewer components. Three components were extracted differentiating three action types: ‘actions to adapt tasks and setting’ (Cronbach’s *α* = 0.91; example: ‘Take away duties from employee’), ‘actions to involve experts’ (Cronbach’s *α* = 0.80; example: ‘Involve HR for the employee’), and ‘social support actions’ (Cronbach’s *α* = 0.89; example: ‘Draw up a concrete action plan together with employee’). A table with the items per component is given in Supplementary Table 4. A sum score was calculated for each component and divided by the number of items. This resulted in a score reflecting the possibility for implementing each action type. The variables representing the possibility for implementing actions are referred to by their component names or ‘action type’ hereafter.

#### Covariates

Covariates were selected based on previous research. Age (six options: < 30, 30–39, 40–49, 50–59, 60–69, > 70 years) and gender (female, 1; male, 0) have previously been associated with stigma [27, 39]. Across Swedish industrial sectors (white, 1; blue, pink, other, 0) gender and education are unevenly distributed, and both factors are associated with stigmatizing attitudes [42, 43]. Therefore, we also included gender distribution of employees (about equal proportions/60% or more females, 0; 60% or more males, 1). Organizational size (seven options: 1–9, 10–49, 50–249, 250–999, 1000–4999, > 5000 employees) was included because it constitutes preconditions for managers’ actions.

### Analysis

Analyses were performed using SPSS (version 28.0, IBM, Armonk, NY) and a significance level of 0.01 was chosen for all analyses to account for multiple testing [44]. Descriptive analysis of all variables was performed, and percentages are reported. Multicollinearity was checked for all multiple linear regression models. Due to considerable skewness, log base 10 transformation was applied to the number of actions variable [45]. The transformed variable was used for the regression analyses.

To answer the first research question, multiple linear regression models were performed with the number of actions as the dependent variable. Model one included all covariates, and models two to five included one stigma layer (personal, employee, collegial, organizational), respectively. The last model comprised all variables. R^2^, ΔR^2^, and standardized β are reported.

The second research question was answered using multiple linear regression models, following the same sequence of model building as used for the first research question. This was repeated once with each action type (actions to adapt tasks and setting; actions to involve experts; social support actions) as the dependent variable. R^2^, ΔR^2^, and standardized β are reported.

## Results

### Sample and Descriptive Statistics

Compared to the study sample (*n* = 2769), the internal dropout (*n* = 709) represented more managers in micro companies (1–9 employees), fewer managers in the pink sector, and fewer managers with a postgraduate degree (see Supplementary Fig. 1).

The descriptive statistics of all variables and covariates are presented in Table 1. Managers within the study sample assessed that on average 17.44 different actions were available to them to prevent the SA of an employee with MHPs. For the actions to adapt tasks and setting, the mean of the possibility to implement this action type was between ‘possible to a certain extent’ (2) and ‘possible to a great extent’ (3). The mean scores of the possibility to implement actions to involve experts and social support actions were slightly higher than ‘possible to a great extent’ (3).

**Table 1:** Descriptive statistics of the study sample (*n* = 2769)

Variables	Answer options	Proportion (%)			Number^a^
*Covariates*					
Age (years)	< 30	0.6			2762
	30–39	9.9			
	40–50	29.9			
	50–59	39.0			
	60–69	17.4			
	> 70	3.3			
Gender	Female	21.2			2761
	Male	78.8			
Organizational size (no. of employees)	1–9	25.3			2769
	10–49	20.4			
	50–249	16.0			
	250–999	11.1			
	1000–4999	12.7			
	5,000 +	14.5			
Industrial sector	White	31.6			2769
	Blue	33.5			
	Pink	20.5			
	Other	14.4			
Gender distribution of subordinates (male)	60% or more men	54.6			2765
	Similar proportions	23.9			
	60% or more women	21.3			
	Mean	SD	Range	Skewness	Number^a^
*Dependent*					
Number of actions	17.44	3.09	0–20	− 1.909	2616
Number of actions (transformed)^b^	0.42	0.34	0–1.3	0.235	2616
Actions to adapt tasks and setting	2.57	0.68	1–4	0.092	2681
Actions to involve experts	3.16	0.91	1–4	− 0.751	2689
Social support actions	3.25	0.64	1–4	− 0.778	2686
*Independent*					
Personal stigmatizing attitudes	29.24	8.68	12–72	0.567	2633
Employee stigma	2.12	0.88	1–5	0.772	2432
Collegial stigma	1.89	1.03	1–5	1.128	2574
Organizational stigma	1.99	1.04	1–5	0.951	2656

*SD* standard deviation

^a^Number changes per variable due to missing values or ‘no opinion’ answers

^b^Variable was transformed using a logarithm of base 10 to adjust for skewness

Moderate correlations (> 0.30) [46] were found among all action types and stigma layer variables, but correlation coefficients were below the threshold for multicollinearity (< 0.80) [47] (Supplementary Table 5).

### Stigma Layers and their Association with the Number of Actions

The results of the regression model with the number of actions as the dependent variable are presented in Table 2. In all models, the stigma layers were significantly and negatively related to the number of actions available to the managers, implying more stigma was related to a smaller number of actions available in the workplace. The contribution to the explained variance ranged from 5.2% to 11.0% in each model with one stigma layer. Overall, the explained variance ranged from 13.1% to 25.7%, with the largest explained variance in the model including all stigma layers. In the final model, employee stigma was no longer significant, personal stigmatizing attitudes contributed 5%, and collegial and organizational stigma together contributed 4.3% to the explained variance.

**Table 2:** Results of the multiple linear regression analyses for ‘number of actions (transformed)’ as the dependent variable

	Variables	Model 1 (*N* = 2599)		Model 2 (*N* = 2520)		Model 3 (*N* = 2298)		Model 4 (*N* = 2428)		Model 5 (*N* = 2505)		Model 6 (*N* = 2163)	
		ΔR^2^	β	ΔR^2^	β	ΔR^2^	β	ΔR^2^	β	ΔR^2^	β	ΔR^2^	β
1	Gender (female)		− 0.051*		− 0.099**		− 0.061*		− 0.054*		− 0.053*		− 0.095**
	Age		0.039		0.041		0.044		0.048		0.031		0.050*
	Industrial sector (white)		0.184**		0.174**		0.173**		0.169**		0.177**		0.161**
	Organizational size		0.324**		0.306**		0.348**		0.329**		0.354**		0.330**
	Gender distribution subordinates (male)	0.132**	0.018	0.077**	− 0.15	0.146**	0.007	0.133**	− 0.006	0.149**	− 0.001	0.134**	0.000
2	Personal stigmatizing attitudes			0.110**	− 0.288*							0.050**	− 0.236**
3	Employee stigma					0.052**	− 0.232**						− 0.050
4	Collegial stigma							0.059**	− 0.246**				− 0.104**
5	Organizational stigma									0.056**	− 0.239**	0.043*^a^	− 0.089**
	Total adjusted R^2^	0.131**		0.216**		0.179**		0.187**		0.187**		0.257**	

*Significant with a *p* value < 0.01

**Significant with a *p* value < 0.001

^a^This ΔR^2^ value indicates the change in variance explained of employee, collegial, and organizational stigma combined

### Stigma Layers and their Association with the Possibilities for Implementing Actions

The results of the regression model with actions to adapt tasks and setting as the dependent variable are presented in Table 3. In all models, the stigma layers were significantly and negatively related to actions to adapt tasks and setting. The contribution to the explained variance ranged from 7% to 11% in each model with one stigma layer. Overall, the explained variance ranged from 7.9% to 25.1%, with the largest explained variance in the model including all stigma layers. In the final model, employee stigma was no longer significant, personal stigmatizing attitudes contributed 7.3%, and collegial and organizational stigma together contributed 5.6% to the explained variance.

**Table 3:** Results of the multiple linear regression analyses for ‘actions to adapt tasks and setting’ as the dependent variable

	Variables	Model 1 (*N* = 2663)		Model 2 (*N* = 2575)		Model 3 (*N* = 2348)			Model 4 (*N* = 2482)			Model 5 (*N* = 2559)			Model 6 (*N* = 2204)	
		ΔR^2^	β	ΔR^2^	β	ΔR^2^	β		ΔR^2^		β	ΔR^2^		β	ΔR^2^	β
1	Gender (female)		− 0.036		− 0.094**			− 0.049		− 0.036			− 0.037			− 0.089**
	Age		0.029		0.025			0.022		0.032			0.013			0.022
	Industrial sector (white)		0.252**		0.241**			0.245**		0.233**			0.247**			0.221**
	Organizational size		0.136**		0.108**			0.160**		0.143**			0.168**			0.134**
	Gender distribution subordinates (male)	0.080**	− 0.034	0.076**	− 0.029	0.085**		− 0.001	0.073**	− 0.015		0.087**	− 0.016		0.071**	− 0.014
2	Personal stigmatizing attitudes			0.110**	− 0.339**										0.073**	− 0.286**
3	Employee stigma					0.072**		− 0.274**								− 0.060
4	Collegial stigma								0.070**	− 0.267**						− 0.106**
5	Organizational stigma											0.077**	− 0.282**		0.056**^a^	− 0.111**
	Total adjusted R^2^	0.079**		0.192**		0.151**			0.149**			0.158**			0.251**	

*Significant with a *p* value < 0.01

**Significant with a *p* value < 0.001

^a^This ΔR^2^ value indicates the change in variance explained of employee, collegial, and organizational stigma combined

The results of the regression model with actions to involve experts as the dependent variable are presented in Table 4. In all models, the stigma layers were significantly and negatively related to actions to involve experts. The contribution to the explained variance were low, ranging from 1.7% to 3.6% in each model with one stigma layer. Overall, the explained variance ranged from 21% to 27.4%, with the largest explained variance in the model including all stigma layers. In the final model, employee and organizational stigma were no longer significant, personal stigmatizing attitudes contributed 2.3%, and collegial stigma 2.5% to the explained variance.

**Table 4:** Results of the multiple linear regression analyses for ‘actions to involve experts’ as the dependent variable

	Variables	Model 1 (*N* = 2672)		Model 2 (*N* = 2587)		Model 3 (*N* = 2357)		Model 4 (*N* = 2493)		Model 5 (*N* = 2568)		Model 6 (*N* = 2216)	
		ΔR^2^	β	ΔR^2^	β	ΔR^2^	β	ΔR^2^	β	ΔR^2^	β	ΔR^2^	β
1	Gender (female)		− 0.029		− 0.060**		− 0.040		− 0.033		− 0.031		− 0.065**
	Age		− 0.018		− 0.017		− 0.020		− 0.012		− 0.025		− 0.015
	Industrial sector (white)		0.014		0.016		0.013		0.012		0.015		0.017
	Organizational size		0.457**		0.446**		0.468**		0.459**		0.480**		0.458**
	Gender distribution subordinates (male)	0.212**	− 0.007	0.203**	− 0.004	0.219**	0.012	0.212**	0.001	0.231**	0.009	0.208**	0.005
2	Personal stigmatizing attitudes			0.036**	− 0.195**							0.023**	− 0.160**
3	Employee stigma					0.017**	− 0.132**						0.044
4	Collegial stigma							0.036**	− 0.190**				− 0.155**
5	Organizational stigma									0.024**	− 0.157**	0.025**^a^	− 0.050
	Total adjusted R^2^	0.210**		0.253*		0.220**		0.235**		0.237**		0.274**	

*Significant with a *p* value < 0.01

**Significant with a *p* value < 0.001

^a^This ΔR^2^ value indicates the change in variance explained of employee, collegial, and organizational stigma combined

The results of the regression model with social support actions as the dependent variable are presented in Table 5. In all models, the stigma layers were significantly and negatively related to social support actions. The contribution to the explained variance ranged from 9.4% to 14.1% in each model with one stigma layer. Overall, the explained variance ranged from 3.2% to 24.6%, with the largest explained variance in the model including all stigma layers. In the final model, personal stigmatizing attitudes contributed 8.9%, and employee, collegial, and organisational stigma together 7% to the explained variance.

**Table 5:** Results of the multiple linear regression analyses for ‘social support actions’ as the dependent variable

	Variables	Model 1 (*N* = 2668)		Model 2 (*N* = 2580)		Model 3 (*N* = 2354)		Model 4 (*N* = 2489)		Model 5 (*N* = 2568)		Model 6 (*N* = 2213)	
		ΔR^2^	β	ΔR^2^	β	ΔR^2^	β	ΔR^2^	β	ΔR^2^	β	ΔR^2^	β
1	Gender (female)		0.077**		0.014		0.072**		0.079**		0.082**		0.028
	Age		− 0.012		− 0.009		− 0.009		− 0.007		− 0.026		− 0.003
	Industrial sector (white)		0.117**		0.109**		0.113**		0.104**		0.115**		0.095**
	Organizational size		0.096**		0.065**		0.121**		0.093**		0.122**		0.092**
	Gender distribution subordinates (male)	0.034**	− 0.050	0.018**	− 0.041	0.033**	− 0.010	0.027**	− 0.028	0.038**	− 0.029	0.018**	− 0.020
2	Personal stigmatizing attitudes			0.141**	− 0.384**							0.089**	− 0.316**
3	Employee stigma					0.097**	− 0.318**						− 0.096**
4	Collegial stigma							0.100**	− 0.319**				− 0.131**
5	Organizational stigma									0.094**	− 0.312**	0.070**^a^	− 0.085*
	Total adjusted R^2^	0.032**		0.176**		0.125**		0.129**		0.124**		0.246**	

*Significant with a *p* value < 0.01

**Significant with a *p* value < 0.001

^a^This ΔR^2^ value indicates the change in variance explained of employee, collegial, and organizational stigma combined

## Discussion

Using a video vignette design, this study investigated the association between managers’ personal stigmatizing attitudes and contextual stigma in the workplace (employee, collegial and organizational) with managers’ possibilities to implement actions to prevent SA of employees with MHPs. Based on principal component analysis, 20 items representing managerial actions were grouped into three action types: (1) actions to adapt tasks and setting; (2) actions to involve experts; and (3) social support actions. Findings indicate both personal stigmatizing attitudes and contextual stigma are associated with a smaller number of actions available to managers and less possibilities to implement actions of any type. These are the first findings illustrating the explanatory value of contextual stigma for the number of actions and possibilities to implement actions that are available to managers to prevent SA in employees with MHPs.

Personal stigmatizing attitudes and contextual stigma contributed similarly to the explained variance of the reported number of actions, suggesting personal stigmatizing attitudes and contextual stigma are separate but equally important factors in reducing managerial action. It has previously been shown that managers working in companies with unsupportive mental health disclosure norms report higher levels of stigma [27], illustrating how different stigma layers (organizational and personal here) might influence one another in the workplace setting. Our results further revealed different patterns of association between the stigma layers and the reported possibilities for implementing actions. In line with previous research into determinants of managerial action taking, these findings further emphasize that managerial actions bear distinct characteristics [24, 25] that are affected by different factors in the workplace. The findings of this study substantiate the need for further research into contextual stigma in the workplace, as well as investigations on managerial actions to prevent SA in consideration of distinct action types.

### Stigma and the Number of Actions to Prevent SA of Employees with MHPs

Managers’ perception of the number of actions available to them to prevent SA was associated with all stigma layers included in this study. Assuming the presence of public and structural stigma, we suggest two possible mechanisms by which personal stigmatizing attitudes and contextual stigma might shape managers’ assessment of number of actions: resources and knowledge. Within the organization, resources for certain actions need to be allocated (e.g. time, money, staff) to allow for certain actions to be available. This explains the substantial association of organizational size and the industrial sector with the number of actions in our findings. Size and sector can influence the predominantly monetary resources available to an organization, while organizational policies determine how these resources can be utilized for SA prevention. Previous research suggests that stigmatized organizational norms can affect company policies, which in turn can limit the actions available to managers [7, 27]. Further, managers seek out the expertise of their managerial colleagues to gain knowledge on actions available within the organization [48], which makes stigma in this group relevant to the individual manager’s knowledge of preventive actions as corroborated in the current study. Inversely, the strong personal stigmatizing attitudes of a manager might prevent them from seeking out information on preventive actions from their peers, resulting in lower number of actions reported.

### Stigma and Possibilities for Implementing Different Types of Actions

A closer investigation of the association between the stigma layers and managers’ possibilities for implementing actions showed that different stigma layers were associated with different action types. Reported possibilities to implement actions to adapt tasks and setting, referring to the actions that managers could take independently of external help (e.g. changing the working hours for the employee), depended largely on the managers’ personal stigmatizing attitudes. Most of these actions rely on single-instance decisions made by the manager. As this type of action does not rely on other social groups, such as employees or colleagues, managers’ own attitudes serve as the point of reference for evaluating the possibility to implement preventative actions. In line with the association found in this study, managers’ personal stigmatizing attitudes can result in negative evaluations [19] and fewer work accommodations [5, 49]. However, contextual stigma, specifically among managerial colleagues and in the organization, contributed to the explained variance. This could be explained by the mechanism of shared social norms. Public and structural stigma contribute to an unsupportive social environment in which managers see less possibility to implement preventative actions, as they simply might not see a need to do so. Research into workplace bullying demonstrates the significant impact that the managerial and organizational contexts can have on managers’ perceptions when tending to employee needs. Findings suggest that managers are encouraged to disregard employees’ concerns and needs for the benefit of productivity goals [50]. Similarly, public stigma could lead managers to perceive tending to the needs of employees with MHPs as less relevant, which reflects in lower reported possibility to implement actions.

Actions to involve experts (in this study referring to HR, union representatives, or occupational health services) were mainly dependent on organizational size; managers from larger companies reported more possibilities to involve experts. For these actions to be possible, a company must have the necessary resources or established ties with occupational health services for SA prevention. This is more often the case in larger companies [51]. However, in addition to organizational size, personal stigmatizing attitudes and contextual stigma were significantly related to the possibility to involve experts.

Possibilities for social support actions were related to all stigma layers. These actions focus on interaction between managers and employees and in some cases the whole team. The interactive nature of these actions requires the manager to engage with the different social layers to address the possibly stigmatized situation of the employee. Hindrances caused by stigma in these different social layers often have an impact on the RTW efforts of employees that are on SA with a CMD [7, 12, 13]. These efforts also require close collaboration of several social groups within organizations [52]. Stigma among managerial colleagues was most strongly associated. This is the most relevant group of reference to the individual manager, therefore preserving their position within this social group has high priority [50]. Managers might be concerned about becoming a target of stigmatization themselves due to close and continuous interaction with the employee with MHPs [53]. This has been referred to as courtesy stigma [29]. Managers might also be hesitant to expose the employee to stigma from their colleagues in an attempt to prohibit the further strengthening of stigma [7]. Thus, low possibilities for social support actions could be rooted in concerns regarding self-protection and employee protection from stigma.

### Categorizing Distinct Managerial Action Types for SA Prevention

The three types of preventive actions emerging from this study overlap with categories of work accommodations reported by managers in previous studies [24, 25]. Although no agreement on overarching categories of actions can be derived from these studies, the overlapping patterns provide grounds for further research to establish comprehensive categories. The preventive actions were identified in focus groups with Swedish managers and piloted on 12 managers who all agreed on the actions, and when specifically asked, did not suggest any further actions to be added to the list. Thus, we adhered to the Swedish setting, but other managerial actions found in international studies may be missing.

### Methodological Strengths and Limitations

The major limitation of this study was the cross-sectional design and that all measures were collected at the level of the manager only. We could thus not draw conclusions on how managerial attitudes lead to experiences at employee level, and also, the different stigma variables share common method variance. However, the large sample size and the video vignette design are strengths. The study sample consisted exclusively of self-recruited managers working in the Swedish private sector, lowering control over the sample composition. This limits the generalizability of the findings within, and especially outside the private sector. Internal dropout analysis showed the study sample under-represents micro companies and managers with postgraduate education, and over-represents the pink private industrial sector. This has implications for the applicability of our findings. However, the substantial sample size in this study provides a good representation of a wide variety of companies.

In the shown vignettes, employees describe problems with ‘concentration, forgetfulness and doubtfulness’. These symptoms are representative of common mental health problems at work but are not representing the full range of experienced problems. Managers’ assessment of taking action was made in reference to the limited symptoms described in the vignette, limiting the generalizability to other symptoms. Despite the more proactive societal context regarding mental health that Sweden has compared to other EU states [54, 55], stigma was shown to be a relevant factor in the workplace regarding the prevention of SA. Therefore, stigma might be even more relevant in contexts with less mental health awareness.

Although relying on self-reported measures, the survey design was strengthened using industry-specific video vignettes, which are seen as preferable over written vignettes to increase the validity of participants’ answers [56]. Randomization of vignettes showing a female or male employee, and phrasing items with reference points to the company, helped prevent gender and social desirability biases confounding the survey responses. However, the cross-sectional data do not allow for causal conclusions. The contextual stigma measures used in this study were newly created but not validated. Despite their novelty, the items were sufficient to demonstrate meaningful associations of different stigma layers with managers’ possibilities to implement actions.

### Recommendations for Research and Practice

This study presents the first significant findings associating the presence of managers’ personal stigmatizing attitudes and contextual stigma, with lower possibilities to prevent SA of employees with MHPs, which have thus far only been described in qualitative studies [7, 12, 13]. Overall, personal stigmatizing attitudes and contextual stigma could explain up to 16% of the variance in managers’ possibilities to implement actions, emphasizing that further research into the hampering effects of stigma in the workplace could substantially contribute towards a better workplace situation for employees with MHPs. The occurrence of stigma emerges from social contexts, therefore further understanding of stigma could be gained through investigation of organizational factors, such as company size and industrial sector, in connection with mental health stigma.

In practice, destigmatization has been part of workplace interventions in several areas [57–59], however lack of evidence-based programmes and changing target groups have been criticized. Small and medium organizations in particular seem to lack resources to create healthy workplaces as shown by previous research and corroborated by our findings [60]. These organizations would benefit from more tailored intervention approaches that can be implemented with minimal resources. Here, destigmatization might be particularly relevant when seeking to have an impact on social support actions in the workplace. Targeting the social context could help foster supportive work environments, which in turn could empower managers’ sense of agency and responsibility. There are three main techniques for the reduction of stigmatizing attitudes in groups: protest, education, and social contact [61]. Protest highlights inequalities and mistreatment of the stigmatized group, and education increases knowledge and counteracts misinformation. Social contact helps normalize MHPs and makes others understand the reality of living with MHPs. Some meta-analytical evidence suggests social contact is the most effective technique, but too little research has tested the long-term effects of the techniques [57]. One recent workplace intervention using educational and social contact techniques for both employees and managers showed promising results, with moderate destigmatization of employees and managers [58, 59]. Investing in an inclusive and destigmatized working environment can shape employees’ positive or negative experiences when disclosing MHPs. A positive experience in disclosing mental health issues has been associated with receiving more managerial support, increased work capacity, and decreased burnout of the employee [62].

There is a need for more robust and validated scales to expand the field of contextual stigma research. Some scales assessing contextual stigma have been developed, such as a scale to evaluate co-workers’ stigma, however specific to the nursing profession [32]. Therefore, new scales should focus on broader applicability.

## Conclusion

This study clearly demonstrated an association of managers’ personal stigmatizing attitudes as well as contextual stigma on the employee, collegial, and organizational level with managers’ possibilities to prevent SA of employees with MHPs. Different stigma layers were relevant for understanding the number of actions available to managers. Further, all stigma layers were to some extent associated with managers’ possibilities to implement actions to adapt tasks and setting, actions to involve experts, and social support actions for SA prevention. These are the first findings substantiating the association of contextual stigma and prevention of SA, highlighting the importance of investigating MHP prevention in its social context and encouraging the further exploration of the concept of contextual stigma in the workplace.

## Supplementary Information

Below is the link to the electronic supplementary material.

Supplementary file1 (DOCX 254 kb)
