# Supplementary material for: Mental Health Stigma in the Workplace and its Association with Possible Actions of Managers to Prevent Sickness Absence of Employees with Mental Health Problems in the Swedish Private Sector: a Video Vignette Study
Source: J Occup Rehabil. 2024 Jul 11;35(3):533–46. doi: 10.1007/s10926-024-10220-z (PMC12361319; doi:10.1007/s10926-024-10220-z)
Supplement: Supplementary file 1 — Supplementary file1 (DOCX 254 kb) [file 10926_2024_10220_MOESM1_ESM.docx]

Mental health stigma in the workplace and its association with possible actions of managers to prevent sickness absence of employees with mental health problems in the Swedish private sector: a video vignette study

Journal of Occupational Rehabilitation

Sofie Schuller^a,b^ (https://orcid.org/0000-0002-3306-3601), Angelique de Rijk^a^, Linda Corin^c^, Monica Bertilsson^d^ (https://orcid.org/0000-0003-2365-2522)

^a^Department of Social Medicine, Primary Care and Public Health Research Institute, Faculty of Health, Medicine and Life Sciences, Maastricht University, the Netherlands

^b^Department of Public Administration and Sociology, School of Social and Behavioural Sciences, Erasmus University, Rotterdam, the Netherlands

^c^Institute of Stress Medicine, Region Västra Götaland, Gothenburg, Sweden

^d^School of Public Health and Community Medicine, Institute of Medicine, The Sahlgrenska Academy, University of Gothenburg, Gothenburg, Sweden

Short title: Mental health stigma in the workplace and sickness absence prevention

Correspondence: Sofie Schuller, MSc, Department of Public Administration and Sociology, Erasmus University, PO Box 1738, 3000 DR Rotterdam, the Netherlands. Tel: +49 157 71980846. E-mail: [schuller@essb.eur.nl](mailto:schuller@essb.eur.nl)

**Supplementary material**

**
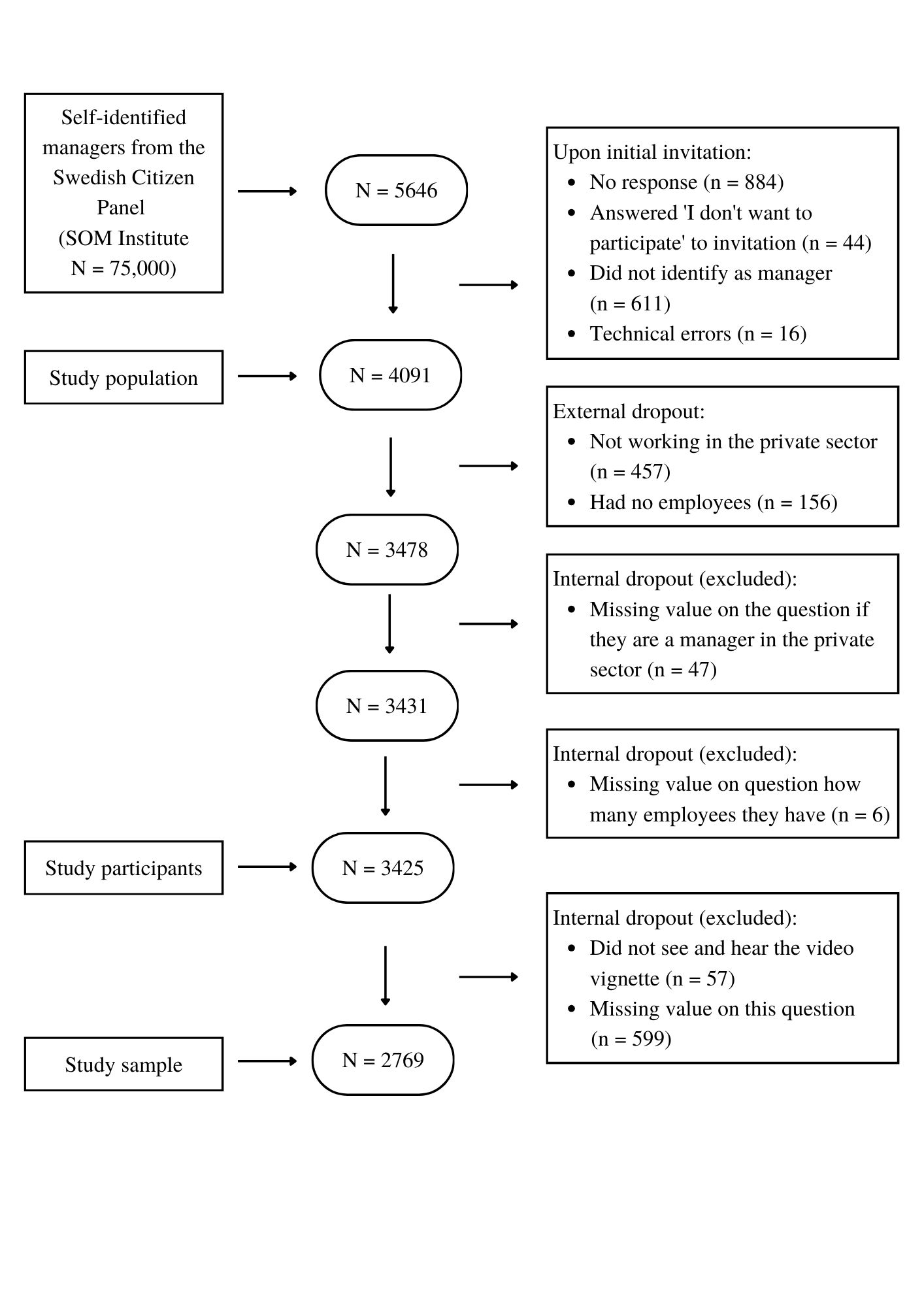
Supplementary Figure 1.** Flowchart from invited participants to study sample.

**Supplementary Table 1.** Revised MSED^a^ items reflecting several MHPs.

| Manager’s affective stigma | Manager’s cognitive stigma | Manager’s behavioural stigma |
| --- | --- | --- |
| (1) I feel very confident when I have to deal with employees with exhaustion, anxiety or depression | (5) If an employee suffers from exhaustion, anxiety or depression, this mainly depends on himself/herself | (9) I would avoid talking with an employee with exhaustion, anxiety or depression in order not to have to deal with their problem |
| (2) It feels frightening having employees with exhaustion, anxiety or depression | (6) Employees with exhaustion, anxiety or depression are a burden on the workplace | (10) I would not employee someone who I knew had suffered from exhaustion, anxiety or depression |
| (3) It’s stressful working with employees with exhaustion, anxiety or depression | (7) Employees who use medication for exhaustion, anxiety or depression should not work | (11) I would make temporary changes to the job to help an exhausted, anxious or depressed employee to recover |
| (4) I feel ill at ease when working with an employee who is exhausted, anxious or depressed | (8) Employees with exhaustion, anxiety or depression can get out of this if only they want to | (12) I would wish that I could get rid of an employee who has suffered from exhaustion, anxiety or depression^b^ |

MSED, Managerial Stigma towards Employee Depression; CMD, common mental disorder.

^a^Martin A. Individual and contextual correlates of managers' attitudes toward depressed employees. Hum Resour Manage. 2010;49(4):647–668. <https://doi.org/10.1037/t49300-000>

^b^Item was adapted to the Swedish legislative context and used in a previous study (Mangerini I, Bertilsson M, de Rijk A, et al. Gender differences in managers' attitudes towards employees with depression: a cross-sectional study in Sweden. BMC Public Health. 2020;20:1744. <https://doi.org/10.1186/s12889-020-09848-2>.

**Supplementary Table 2.** Industrial sectors and their associated work fields.

| Industrial sector | Field |
| --- | --- |
| White | IT, information and communications activities |
|  | Finance and insurance |
|  | Public administration and defence |
|  | Legal, economic, scientific and technological activities |
|  | Property |
| Blue | Agriculture, foster, fishing |
|  | Mining and quarrying |
|  | Manufacturing industry |
|  | Construction and craftsmanship |
|  | Provision of electricity, heat, water, sewage, waste |
|  | Transport and warehousing |
| Pink | Trade/commerce |
|  | Hospitality |
|  | Education |
|  | Health care, social services |
|  | Rental, property service, travel, and other support services |
|  | Gainful employment in households and other service operations |
| Other | Culture entertainment, recreation |
|  | Other type of activity |

**Supplementary Table 3.** Script of video vignettes per industrial sector (each vignette is one minute).

| Industrial sector | Vignette script |
| --- | --- |
| White | *Manager*: You wanted to talk about something.  *Employee*: Yesterday when I was going to finish that report, I got stuck... I read through our notes several times but... I couldn't get it right. I have such a hard time getting it right. It just doesn't work. I've already had to ask the others loads of times.  And it’s not just that, I don't remember things. Even things I've done a hundred times before, I can just forget how to do them.  And I... Well, the other day, I couldn't remember how to get into the staff system on the computer.  And I forget things I’m supposed to do, just like that... I think you already know that I forgot about some meetings last week that I’d promised to arrange.  *Manager*: Yes, I heard about that.  *Employee*: Well, I don't know what to do. I find it so hard that I can't trust myself, and that I miss things all the time. I mean... maybe I need to take sick leave? |
| Blue | *Manager*: You wanted to talk about something.  *Employee*: Yes, yesterday when I went to start up our new machine, I got stuck... I read the manual several times but... I couldn’t get it right. I have such a hard time getting it right. It just doesn't work. I've already had to ask the others loads of times.  And it’s not just that, I don't remember things. Even things I've done a hundred times before, I can just forget how to do. The other day... I didn't know how to get into the staff system on the computer.  And I forget things I'm supposed to do... just... like that. I think you may already know that I... forgot about the tools last week which I’d promised to fix.  *Manager*: Yes, I heard about that.  *Employee*: Well, I don't know what to do. I find it so hard that I can't trust myself, and that I miss things all the time. I mean... maybe I need to take sick leave? |
| Pink | *Manager*: You wanted to talk about something?  *Employee*: Yesterday, when we were looking after the new participants, I got stuck... I tried several times to get into the conversation but... no, I couldn't get it right. And I have such a hard time getting it right. It just doesn't work. I've already had to ask the others loads of times.  And it’s not just that, I don't remember things. Even things I've done a hundred times before, I can just forget... how to do them. The other day I didn't know how to get into the staff system on the computer.  I forget about things I’m supposed to do, just like that... I think you already know that I forgot about both Sara and Simon last week, which I’d promised to address.  *Manager*: Yes, I heard about that.  *Employee*: Well, I don't know what to do. I find it so hard that I can't trust myself, and that I miss things all the time. I mean... maybe I need to take sick leave? |

**Supplementary Table 4.** Types of actions: items per component based on principal component analysis

| Component 1: **Actions to adapt tasks and setting** | Component 2: **Actions to involve experts** | Component 3: **Social support actions** |
| --- | --- | --- |
| 1) Take away duties from employee | 1) Involve HR for the employee | 1) Support employee actively in everyday work (e.g. via test message reminder) |
| 2) Assign employee easier work duties | 2) Involve corporate healthcare or equivalent for the employe | 2) Hold regular, planned follow-up meetings with employee |
| 3) Give employee more time to carry out work duties | 3) Contact employee’s union and/ or safety representative | 3) Draw up a concrete action plan together with employee |
| 4) Offer employee more breaks during the work day |  | 4) Draw up checklist together with employee to help them cope with work duties that they normally have to carry out completely independently |
| 5) Give employee access to a calm space for relaxation and recovery |  | 5) By agreement with employee, talk with the work group about their situation |
| 6) Change working hours for employee |  | 6) Appoint colleague to provide support for the employee |
| 7) Allow employee to work from home |  |  |
| 8) Assign employee’s work duties to other employees |  |  |
| 9) Increase staffing to compensate for employee’s reduced work capacity |  |  |
| 10) Reassign employee to other work |  |  |
| 11) Review the workplace in general, and make changes that benefit both the employee and the work group |  |  |

**Supplementary Table 5.** Correlation matrix of all variables of interest.

| Variables | Age | Gender (female) | Organizational size | Industrial sector (white) | Gender distribution of subordinates (male) | Number of actions (transformed) | Actions to adapt tasks and setting | Actions to involve experts | Social support actions | Personal stigmatizing attitudes | Employee stigma | Collegial stigma | Organizational stigma |
| --- | --- | --- | --- | --- | --- | --- | --- | --- | --- | --- | --- | --- | --- |
| Age | 1 | −0.099** | −0.198** | −0.062** | −0.022 | −0.49* | −0.008 | −0.107** | −0.044* | 0.042* | −0.094** | −0.030 | −0.095** |
| Gender (female) |  | 1 | 0.024 | −0.029 | −0.037 | −0.029 | 0.037 | 0.012 | 0.092 | −0.183** | −0.025 | 0.005 | 0.027 |
| Organizational size |  |  | 1 | −0.036 | 0.016 | 0.259** | 0.123** | 0.459** | 0.096** | −0.096** | 0.138** | 0.064** | 0.137** |
| Industrial sector (white) |  |  |  | 1 | −0.079** | 0.153** | 0.243** | 0.000 | 0.113** | −0.045* | −0.043* | −0.089** | −0.041* |
| Gender distribution of subordinates (male) |  |  |  |  | 1 | −0.048* | −0.066** | 0.006 | −0.054** | 0.018 | 0.065** | 0.035 | 0.037 |
| Number of actions (transformed) |  |  |  |  |  | 1 | 0.712** | 0.547** | 0.567** | −0.310** | −0.219** | −0.263** | −0.222** |
| Task and setting oriented strategies |  |  |  |  |  |  | 1 | 0.377** | 0.645** | −0.338** | −0.262** | −0.281** | −0.271** |
| Actions to involve experts |  |  |  |  |  |  |  | 1 | 0.435** | −0.227** | −0.062** | −0.160** | −0.086** |
| Social support actions |  |  |  |  |  |  |  |  | 1 | −0.397** | −0.310** | −0.325** | −0.298** |
| Personal stigmatizing attitudes |  |  |  |  |  |  |  |  |  | 1 | 0.212** | 0.206** | 0.210** |
| Employee stigma |  |  |  |  |  |  |  |  |  |  | 1 | 0.671** | 0.720** |
| Collegial stigma |  |  |  |  |  |  |  |  |  |  |  | 1 | 0.682** |
| Organizational stigma |  |  |  |  |  |  |  |  |  |  |  |  | 1 |
| *Two-tailed significance at the 0.05 level. | | | | | | | | | | | | | |
| **Two-tailed significance at the 0.01 level. | | | | | | | | | | | | | |
